# Balance Performance as Observed by Center-of-Pressure Parameter Characteristics in Male Soccer Athletes and Non-Athletes

**DOI:** 10.3390/sports5040086

**Published:** 2017-11-08

**Authors:** Lara A. Thompson, Mehdi Badache, Steven Cale, Lonika Behera, Nian Zhang

**Affiliations:** 1 Biomedical Engineering Program, Department of Mechanical Engineering, School of Engineering and Applied Sciences, University of the District of Columbia, 4200 Connecticut Ave. NW, Washington, DC 20008, USA; 2 Department of Mechanical Engineering, School of Engineering and Applied Sciences, University of the District of Columbia, 4200 Connecticut Ave. NW, Washington, DC 20008, USA; dabcha1991@gmail.com (M.B.); steven.cale12@gmail.com (S.C.); lonika.behera@liveudc.onmicrosoft.com (L.B.); 3 Department of Electrical & Computer Engineering, School of Engineering and Applied Sciences, University of the District of Columbia, 4200 Connecticut Ave. NW, Washington, DC 20008, USA; nzhang@udc.edu

**Keywords:** balance, injury prevention, performance, athletes, physical activity, center-of-pressure

## Abstract

Static balance has a relevant influence on athletic performance as well as on reducing the risk of injury. The main goal of this study was to assess soccer athlete versus non-athlete balance performance via displacement and velocity parameters extracted from the center-of-pressure (COP) position time series. In order to accomplish our goal, we investigated standing balance in two male groups with unimpaired balance: non-athletes (*n* = 12) and collegiate varsity soccer athletes (*n* = 12). In order to make the standing balancing task more or less difficult, we altered participant base-of-support, as well as vision, yielding static (quiet stance) test conditions increasing in difficulty. From the COP position time series, displacement and velocity parameters were computed and plotted as a function of increasing test condition difficulty level. COP parameters showed steeper increases with increased test difficulty in non-athletes compared to athletes; this demonstrated athletes’ better ability to control their balance. We concluded that balance performance could be characterized via COP displacement and velocity response curves. This study lends new insights into how COP parameters can be utilized to determine and characterize improvements in balance between un-impaired subject populations (athletes versus non-athletes).

## 1. Introduction

How balance is affected—in particular, how it can be improved—by various activities and interventions, including athletic training, are common concerns [1,2,3,4,5,6,7,8,9,10,11,12,13,14,15,16,17,18,19,20]. It is common knowledge that soccer is perhaps one of the most popular sports worldwide, and balance is critical to this group of athletes towards: (1) competitive performance (e.g., stability of supporting foot-turns during shooting and maneuvering); and (2) reduction of injury-risk. However, in order to prevent injuries, one must first understand how training can impact (improve) imbalance and improve proprioception (i.e., the ability of one to perceive their body segments relative to one another to stabilize their center-of-mass). Training and exercises that improve proprioception may improve one’s postural capabilities, in particular, one’s balance. However, when subjected to a particular training plan and/or interventional aid/method, distinct empirical measures are needed to quantify, characterize and define one’s improvements. In particular, more sensitive measures of balance are needed for populations that are unimpaired (e.g., soccer athletes and non-athletes) that wish to improve their balance to prevent injury and/or to predict (balance) performance [16,17,18,19,20]. As we discuss here, postural performance may be characterized by the ability to reduce postural sway, as observed by the center-of-pressure (COP)-derived parameters shown here.

The COP represents body motion in space as detected at the interface between the feet and the ground. The COP position represents the location of the (resultant) vertical ground reaction force vector [1,2]. This is used as a means to quantify one’s balance: greater displacement of the COP position could mean greater instability. However, balance performance is most commonly assessed via qualitative measures, for example, the Berg Balance Scale (BBS), Activities-Specific Balance Confidence (ABC) Scale (as in [3,4,5]), Star Excursion Balance Test (SEBT) (as in [6,7,8]), and Balance Error Scoring System (BESS) (as in [8,9]), for various populations, including athletes. 

Several studies have reported that soccer training strongly influences balance abilities [10,11,12,13,14,15,16]. One study, conducted by Butler et al. [17], utilized the lower quarter Y balance test (foot reaches in multiple directions) to observe balance dynamic balance abilities among male high school, collegiate, and professional soccer players. Dynamic balance performance (based solely on foot-reach) varied with competition level. Ricotti et al. [18] reported soccer players’ performance in terms of maximum vertical jump height (to determine maximal leg strength), contact time (to assess acyclic rapidity, or quickness), static & dynamic balance, and reaction times. Bressel et al. [8] studied the effects of soccer, gymnastics, and basketball on balance stability in female athletes. However, it is possible that the BESS and SEBT assessments used may have been too insensitive to fully capture the differences between the unimpaired, athletic groups. The above studies lead one to question whether the assessments used truly captured the (balance) differences between the groups of soccer athletes. 

Pallard et al. [19] also studied soccer players’ balance and compared postural performance and postural strategy between soccer players at different levels of competition (national vs regional athletes) for unipedal stance. Instead of qualitative measures, the COP was measured (surface area, velocity, and spectra) for both groups, and it was observed that COP surface areas and velocity were greater for regional soccer players than for national soccer players, indicating greater instability. A set back of this study was that, instead of a forceplate, only 3 strain gauges were used to measure the force data leading to low resolution. A second setback was that only unipedal stance was examined during their study. Gerbino et al. [20] compared standing balance between female collegiate dancers and soccer players. In comparison to the other studies above, the advantages to this study were that they measured the ground reaction COP position shifts based on a high-resolution (Tekscan) forceplate, and then they quantified the COP sway index, path length, and sway velocity. In 5 of the 20 balance tests, they found that dancers had significantly better balance than the soccer players. However, this study did not compare the difference between non-athlete subjects and athlete groups, nor the roles of vision and support base for the aforementioned groups. 

Empirical measures for quantifying balance and, perhaps more importantly, balance improvements and the effects of training, are important for injury prevention as well as rehabilitation. Velocity and displacement information derived from the COP position time series could prove more sensitive and informative than the commonly used qualitative measures (e.g., ABC scale and BESS test) discussed above. 

The purpose of this study was to assess male varsity collegiate soccer athlete versus male non-athlete balance performance (via COP displacement and velocity parameters) during standing balancing tasks for which we altered base-of-support, as well as vision. Our hypotheses were that: (1) athlete versus non-athlete balance performance could be defined via displacement and velocity parameters derived from the COP (i.e., athletes would have slower COP velocities and smaller COP displacements for more difficult quiet stance conditions than non-athletes); and (2) the effects of training on balance performance could be characterized and clearly observed within COP-derived displacement and velocity response curves (i.e., in general, steeper curves for non-athletes compared to athletes as quiet standing task difficulty increased).

## 2. Materials and Methods

We describe our methodology and study design, in terms of participants, experimental protocol, and equipment and data analysis, denoted by the sub-headings below. All human subject testing was conducted within the University of the District of Columbia’s (UDC’s) Center for Biomechanical & Rehabilitation Engineering Laboratory. All subjects gave their informed consent for inclusion before they participated in the study. Further, the study was conducted in accordance with the Declaration of Helsinki, and was approved by UDC’s Institutional Review Board (Project identification code: 540869-1).

### 2.1. Participants

Both athlete and non-athlete participants performed quiet stance balancing tasks in the absence of external perturbations. This study consisted of two male test groups: (1) a (control) group of 12 “non-athletes”, who exercised with below-moderate activity (e.g., walking, minimal activity, or no activity), less than 2 sessions per week, each week; (2) a group of 12 “athletes”, who participated in varsity collegiate soccer at UDC and followed an identical training regimen. Both groups were young, male adults (athletes: 23 ± 2 years old; non-athletes: 27 ± 3 years old), free from sensory neuropathies, were able to ambulate without assistance, and had not suffered from a fall-related injury and/or concussion in the past 5 years. 

### 2.2. Protocol

It is well known that inputs to the visual, somatosensory and vestibular systems are used for postural control, and further, base-of-support impacts one’s balance [1]. For our test battery, we varied visual cues, and also stance width, to make the standing task more and more difficult. For standing balance, subjects were tested for eyes-open/eyes-closed (receiving/not receiving visual system input) for both wide/tandem foot placement conditions (wide foot placement and front-to-back foot placement, respectively) leading to four test conditions: (1) wide/eyes-open, (2) wide/eyes-closed, (3) tandem/eyes-open, (4) tandem/eyes-closed. For each condition, four 30-second data sets were recorded, with a brief rest in between. 

### 2.3. Equipment and Data Analysis

**Figure 1:**
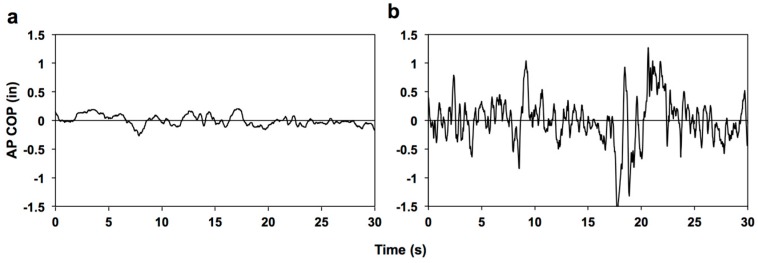
Example AP COP position time series traces for a non-athlete subject: (**a**) eyes-open/wide test condition (easiest) and (**b**) eyes-closed/tandem test condition (most difficult).

During our experiments, subjects’ standing balance mediolateral (ML, or side-to-side) and anterior-posterior (AP, or front-to-back) COP position traces were measured using a Tekscan Forceplate Walkway (Boston, MA, USA) and data acquisition involved the use of the Tekscan Forceplate Software (Tekscan Inc, Boston, MA, USA), installed on a Dell PC computer within the lab. Each subject’s ground reaction force data was acquired at a rate of 50 Hz. The Tekscan software allowed for the raw ground reaction force and COP position data to be exported. Shifts in COP position, or changes in the location (position) of resultant vertical ground reaction force vector, as a function of time were recorded. Non-athlete, AP COP (example) position traces are shown in Figure 1a,b.

MATLAB software (MathWorks, R 2014a, Natick, MA, USA) was used for all post-processing of the data, including statistical analysis. From the COP position trace as a function of time was post-processed to compute displacement, velocity, parameters [21]. Parameters were computed for AP as well as ML COP position traces. In particular: mean displacement (MD), root-mean-squared (RMS), peak-to-peak range of displacement (MAXD), root-mean-square velocity (RMSV), and mean velocity (MV) of the COP. The formulas used to calculate these parameters are provided in the Appendix A. 

For each group, average values for each parameter were computed from the 192 usable trials (total) per group. The results were pooled for the non-athlete group and the athlete group for each test condition. For each of the four test conditions, there were 48 trials each per group. In terms of statistical analysis, for each group and for each test condition, the trials were averaged and the standard errors computed for the above parameters. Significant differences were observed as *p*-values < 0.05 and assessed using t-tests for equal sample size, unequal variance. 

## 3. Results

### 3.1. AP and ML COP Displacement and Velocity Parameters as a Function of Quiet Stance Test Condition

**Figure 2:**
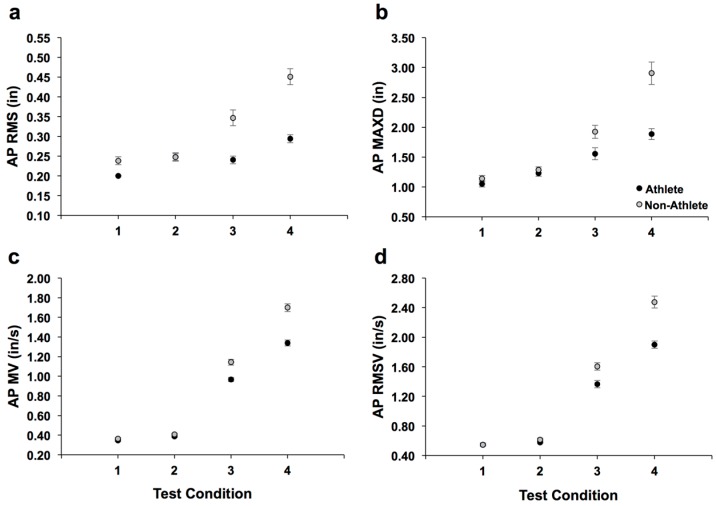
Mean values and standard errors for AP COP parameters (**a**–**d**) for athletes (filled circles) and non-athletes (open circles). For each condition, there were a total of 48 trials. Test conditions: 1 = eyes-open/wide stance; 2 = eyes-closed/wide-stance; 3 = eyes-open/tandem stance; 4 = eyes-closed/tandem stance.

Here we display characteristics captured by the displacement and velocity parameters extracted from the COP for soccer athlete and non-athlete groups. Plots of means and standard errors are shown in Figure 2a,b for the AP direction for the two displacement parameters (i.e., RMS and MAXD) and two velocity parameters in Figure 2c,d (i.e., MV and RMSV) as a function of test condition. The increases in test condition difficulty (i.e., eyes-open/wide = easiest and eyes-closed/tandem = most difficult) are from left (“1”) to right (“4”) on the x-axis, respectively. 

Table 1 summarizes mean values, degrees-of-freedom, *t*-value, and level of significance (*p*-values) for parameters with significant differences for each test condition in the AP and ML directions. All significant differences in the COP parameters for both AP and ML directions were seen as *decreases* (between athletes compared to non-athletes). Decreases in COP parameters may be interpreted as increases in balance stability. It is important to note that 4 of the 12 non-athletes had great difficulty for the eyes-closed/tandem condition (the most difficult of the four test conditions). These 4 non-athlete participants had repeated stepping (or “falls”) for the eyes-closed/tandem condition, which were not averaged within the data set. However, these trials with stepping were recaptured. 

### 3.2. AP Displacement and Velocity Parameter Characteristics

**Figure 3:**
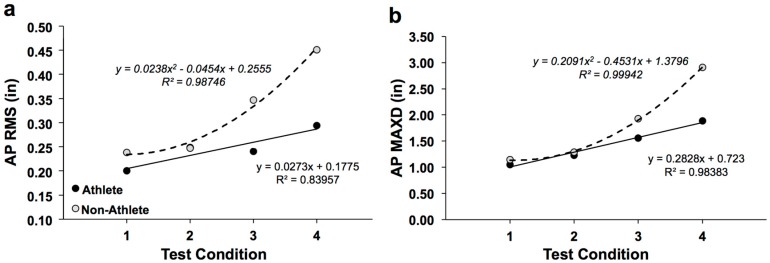
Displacement parameters: (**a**) AP RMS & (**b**) AP MAXD); Trend lines (with R-squared values and equations shown) for athletes (filled circles) and non-athletes (open circles).

**Figure 4:**
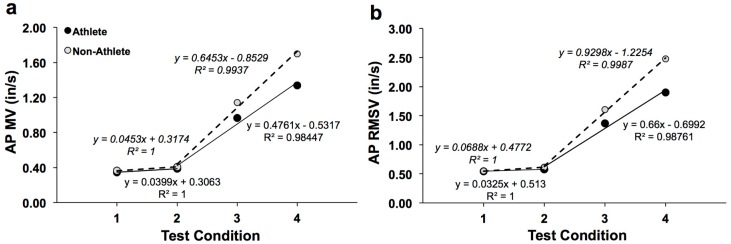
Velocity parameters: (**a**) AP MV & (**b**) AP RMSV; Trend lines (with R-squared values and equations shown) for athletes (filled circles) and non-athletes (open circles).

Figure 3 shows the AP displacement response curves for soccer athletes and non-athletes. AP RMS (Figure 3a) as a function of quiet stance test condition difficulty had a steeper increase (fitted with parabolic trend) for non-athletes, whereas AP RMS appeared to increase with a moderate increase (fitted with linear trend) as a function of increased difficulty, both with high R-squared value fits; this observation was also true for AP MAXD (Figure 3b). For AP MV, and AP RMSV, eyes-open/wide (“1”) and eyes-closed/wide (“2”) were approximately flat (Figure 4a,b). However, linear increases were observed for the two tandem conditions (“3” & “4”) that were more difficult. This was observed in both the athletes and non-athletes; however, for the non-athletes, the slope was steepened (Figure 4a,b).

## 4. Discussion

From our results, we were able to make meaningful comparisons between non-athlete and soccer athlete participants, as well as form general conclusions about the effects of soccer athletic training on balance and postural control.

In our study, observable increases in balance stability (decreases in COP displacement and velocity parameters) were seen in soccer athletes compared to non-athletes. In particular, non-athletes had great difficulty balancing for the test condition where vision was limited and base-of-support was small (i.e., eyes-closed/tandem), with some even losing their balance and stepping. However, all the soccer athletes maintained their balance. Contrary to the non-athlete responses, it was observed that the soccer athletes had decreased COP position and velocity parameters, in particular, for the more difficult test conditions. The decreases seen in COP velocity were interpreted as soccer athletes’ ability to anticipate body position change more effectively than the non-athletes.

Our study allowed for more detailed comparisons than in previous studies which used more qualitative-type assessments, crude forceplate assessments, did not alter vision and/or base-of-support, and/or did not compare athlete versus non-athlete balance performance [16,17,18,19,20]. Further, it is likely that the commonly used assessments (e.g., BESS and SEBT) are too insensitive to fully capture the differences between the unimpaired athletic groups. Here, we aimed to capture (balance) differences between groups of soccer athletes and non-athletes while using a high-resolution forceplate to ultimately obtain COP-based parameters for eyes-open/closed with either wide/tandem stance. Comparing differences in COP characteristics between non-athlete subjects and soccer athlete groups, while altering the roles of vision and support base, were unique aspects posed here. 

It is well known that inputs to the visual, somatosensory and vestibular systems are used for postural control, and further, base-of-support impacts one’s balance [1]. Destabilizing influences, such as gravity, produce external forces on the body that one must counteract in order to remain upright. Quiet standing involves continuous modulation of ankle extensor muscle activity to compensate for one’s spontaneous sway, even in the absence of other external perturbations. The ankle torque needed to maintain upright stance consists of a passive torque and an active torque. The passive torque is related to tension/stiffness produced by muscle tonus and by the stiffness of the surrounding tissue, such as ligaments and tendons, whereas the active torque is produced by sensorimotor integration which modulates/controls muscle contractions. Active torque generation from velocity feedback, in addition to position feedback, can be described in terms of a proportional + derivative (or “PD”) control. Velocity feedback allows prediction of the future condition of a system, and can thus stabilize the body more effectively than a position/proportional controller on its own. Body position and velocity information feed back to the central nervous system during standing, and allow for ankle extensor activity changes in an anticipatory fashion, therefore facilitating effective control for quiet stance (e.g., [22,23])). This ‘model’ for quiet stance control has been used in previous studies of human postural control (e.g., [22,23,24,25]). In our study, we did not model the posture system using a PD controller model, per se. However, the above description assisted us in interpreting the behavioral differences observed between the soccer athletes’ and non-athletes’ COP-derived displacement and velocity parameter results. 

In the soccer athletes, ankle extensor muscle activity and improved anticipatory postural responses are likely contributors to their improved stability. The soccer athletes had undergone rigorous exercise, routine static and dynamic stretching for all training and competitive sessions, foot-eye coordination work (e.g., jumping, running backwards, maneuvering through cones), weight circuits including foam squats, balance ball, jumping on boxes (for injury prevention), and drilling activities, all of which excessively trained their anticipatory postural control and contributed to increased ankle torque capacity. The above activities contributed to the athletes having (1) increased, inherent passive (ankle) torque generation; and (2) increased ability to make use of their sensory information to produce an active torque (e.g., improved interface between neural stimulus and a muscular response). Further, as observed in Table 1, all ML parameters were significantly decreased in the soccer athlete group compared to non-athlete group. Increased ML stability has been previously associated with decreased fall-risk [26]. We interpreted our finding as the soccer athlete group was perhaps less prone to an unintentional fall-related injury than the non-athlete group.

## 5. Conclusions

When subjected to a particular training plan and/or interventional aid/method, distinct empirical measures are needed to quantify, characterize and define changes as well as improvements. This study lends new insights as to how parameters derived from the COP position time series can be utilized to determine and characterize improvements in balance between un-impaired subject populations (athletes versus non-athletes). 

## Figures and Tables

**Table 1:** For each condition, observed means and significant differences between soccer athletes (A) and non-athlete (NA) parameters in AP and ML. Gray text indicates insignificant differences.

Test Condition	Anterior-Posterior (AP)				Mediolateral (ML)			
	1. Eyes-Open/Wide	2. Eyes-Closed/Wide	3. Eyes-Open/Tandem	4. Eyes-Closed/Tandem	1. Eyes-Open/Wide	2. Eyes-Closed/Wide	3. Eyes-Open/Tandem	4. Eyes-Closed/Tandem
**RMS (in**)	**A = 0.20, NA = 0.24** *df* = *86*, *t* = −*2.65*	**A = 0.25, NA = 0.25** *df* = *87*, *t* = *0.11*	**A = 0.24, NA = 0.35** *df* = *78*, *t* = −*4.78*	**A = 0.29, NA = 0.45** *df* = *69*, *t* = −*5.62*	**A = 0.13, NA = 0.16** *df* = *72*, *t* = −*3.47*	**A = 0.15, NA = 0.16** *df* = *86*, *t* = −*0.31*	**A = 0.24, NA = 0.27** *df* = *89*, *t* = −*3.42*	**A = 0.39, NA = 0.45** *df* = *84*, *t* = −*4.02*
**MAXD (in**)	**A = 1.05, NA = 1.14** *df* = *94*, *t* = −*1.26*	**A = 1.23, NA = 1.29** *df* = *94*, *t* = −*0.83*	**A = 1.56, NA = 1.92** *df* = *93*, *t* = −*2.41*	**A = 1.88, NA = 2.91** *df* = *68*, *t* = −*5.62*	**A = 0.77, NA = 0.88** *df* = *92*, *t* = −*1.56*	**A = 0.83, NA = 0.98** *df* = *75*, *t* = −*2.05*	**A = 1.29, NA = 1.45** *df* = *88*, *t* = −*3.00*	**A = 1.94, NA = 2.35** *df* = *75*, *t* = −*4.44*
**MV (in/s)**	**A = 0.35, NA = 0.36** *df* = *91*, *t* = −*1.16*	**A = 0.39, NA = 0.41** *df* = *94*, *t* = −*1.77*	**A = 0.97, NA = 1.14** *df* = *92*, *t* = −*4.85*	**A = 1.34, NA = 1.70** *df* = *77*, *t* = −*7.29*	**A = 0.32, NA = 0.40** *df* = *94*, *t* = −*5.50*	**A = 0.33, NA = 0.42** *df* = *73*, *t* = −*5.68*	**A = 0.66, NA = 0.74** *df* = *91*, *t* = −*4.59*	**A = 1.22, NA= 1.36** *df* = *81*, *t* = −*4.20*
**RMSV (in/s)**	**A = 0.55, NA = 0.55** *df* = *94*, *t* = −*0.56*	**A = 0.58, NA = 0.61** *df* = *88*, *t* = −*1.88*	**A = 1.37, NA = 1.60** *df* = *94*, *t* = −*3.48*	**A = 1.90, NA = 2.47** *df* = *78*, *t* = −*6.32*	**A = 0.49, NA = 0.58** *df* = *94*, *t* = −*3.95*	**A = 0.50, NA = 0.68** *df* = *59*, *t* = −*6.00*	**A = 0.96, NA = 1.05** *df* = *90*, *t* = −*3.32*	**A = 1.72, NA = 1.92** *df* = *75*, *t* = −*3.74*

Insignificant difference***p* < 0.05*****p* < 0.02*****p* < 0.01*****p* < 0.005*****p* < 0.002*****p* < 0.001**

## Appendix A

*Maximum distance (MAXD):*

(A1) MAXD=max(x(i))−min(x(i))

*Root-mean-square of trunk position (RMS):*

(A2) $$RMS=\sqrt{\frac{1}{N}\sum_{i=1}^{N}{\left[x(i)\right]}^{2}}$$

where *x*(*i*) is COP data for sample number “*i*”, *N* = number of samples.

*Mean velocity (MV):*

(A3) $$MV=\frac{1}{N-1}\sum_{i=1}^{N-1}\left|\dot{x}(i)\right|$$

*Root-mean-square velocity (RMSV):*

(A4) $$RMSV=\sqrt{\frac{1}{N-1}\sum_{i=1}^{N-1}{\left[\dot{x}(i)\right]}^{2}}$$

where $\dot{x}(i)$ is derivative of the COP data for sample number “*i*”, *N* = number of samples.
